# 1-(2-Meth­oxy­phen­yl)-1*H*-pyrrole-2,5-dione

**DOI:** 10.1107/S1600536812026888

**Published:** 2012-06-30

**Authors:** Muhammad Sirajuddin, Saqib Ali, M. Nawaz Tahir

**Affiliations:** a Department of Chemistry, Quaid-i-Azam University, Islamabad, Pakistan; b Department of Physics, University of Sargodha, Sargodha, Pakistan

## Abstract

In the title compound, C_11_H_9_NO_3_, the dihedral angle between the meth­oxy­benzene and 1*H*-pyrrole-2,5-dione rings is 75.60 (10)°. The C atom of the meth­oxy group is close to coplanar with its attached ring [deviation = 0.208 (2) Å]. In the crystal, weak aromatic π–π stacking [centroid–centroid separation = 3.8563 (13) Å] occurs between inversion-related pairs of benzene rings.

## Related literature

For a related structure, see: Carroll *et al.*, (2011 ▶).
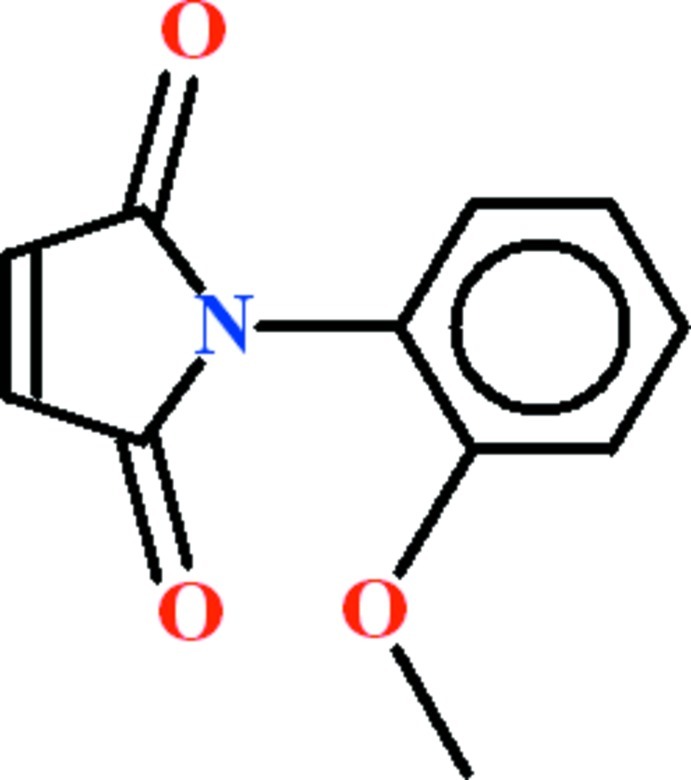


## Experimental

### 

#### Crystal data

C_11_H_9_NO_3_
*M* *_r_* = 203.19
Monoclinic, 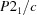

*a* = 12.7018 (15) Å
*b* = 10.2689 (12) Å
*c* = 7.4695 (8) Å
β = 101.067 (7)°
*V* = 956.16 (19) Å^3^
*Z* = 4
Mo *K*α radiation
μ = 0.10 mm^−1^
*T* = 296 K
0.30 × 0.25 × 0.23 mm

#### Data collection

Bruker Kappa APEXII CCD diffractometer
Absorption correction: multi-scan (*SADABS*; Bruker, 2005 ▶) *T*_min_ = 0.969, *T*_max_ = 0.977
7388 measured reflections
1887 independent reflections
1267 reflections with *I* > 2σ(*I*)
*R*_int_ = 0.030

#### Refinement

*R*[*F* ^2^ > 2σ(*F* ^2^)] = 0.041
*wR*(*F* ^2^) = 0.112
*S* = 1.01
1887 reflections
137 parameters
H-atom parameters constrained
Δρ_max_ = 0.11 e Å^−3^
Δρ_min_ = −0.19 e Å^−3^

### 

Data collection: *APEX2* (Bruker, 2007 ▶); cell refinement: *SAINT* (Bruker, 2007 ▶); data reduction: *SAINT*; program(s) used to solve structure: *SHELXS97* (Sheldrick, 2008 ▶); program(s) used to refine structure: *SHELXL97* (Sheldrick, 2008 ▶); molecular graphics: *ORTEP-3 for Windows* (Farrugia, 1997 ▶) and *PLATON* (Spek, 2009 ▶); software used to prepare material for publication: *WinGX* (Farrugia, 1999 ▶) and *PLATON*.

## Supplementary Material

Crystal structure: contains datablock(s) global, I. DOI: 10.1107/S1600536812026888/hb6853sup1.cif

Structure factors: contains datablock(s) I. DOI: 10.1107/S1600536812026888/hb6853Isup2.hkl

Supplementary material file. DOI: 10.1107/S1600536812026888/hb6853Isup3.cml

Additional supplementary materials:  crystallographic information; 3D view; checkCIF report

## Figures and Tables

**Fig. 1.**
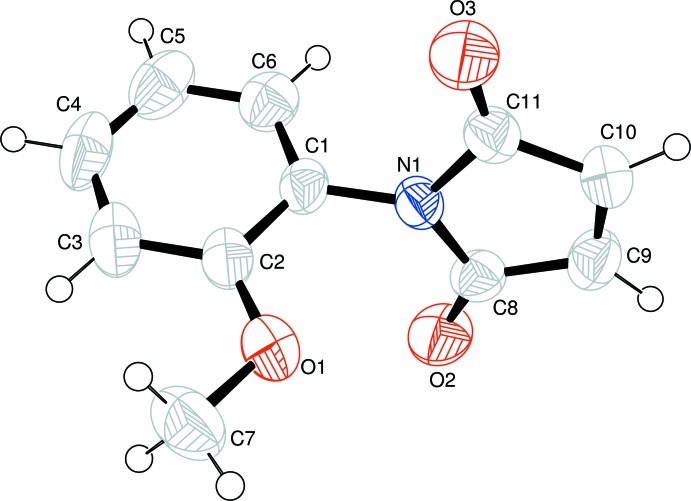
View of the title compound with displacement ellipsoids drawn at the 50% probability level.

## supplementary crystallographic information

### Comment

The title compound (I), (Fig. 1) is present as a fragment of the crystal structure of 4-(2-methoxyphenyl)-4-azatricyclo[5.2.1.0^2,6^]dec-8-ene-3,5-dione (Carroll *et al.*, 2011).

In (I) the methoxybenzene A (C1—C7/O1) and 1*H*-pyrrole-2,5-dione B (C8—C11/N1/O2/O3) are close to planar with r.m.s. deviation of 0.0461 and 0.0201 Å, respectively. The dihedral angle between A/B is 78.22 (5)°.

### Experimental

Equimolar quantities of 2-methoxyaniline and furan-2,5-dione (maleic anhydride) were stirred in acetic acid for 2 h. The solution was kept at room temperature which afforded light yellow prisms after two days.

### Refinement

Twin was found in the data with twin matrix [1, 0, 0.653: 0, -1, 0: 0, 0, -1]. Using the standard techniques, the twin was removed with Basf = 0.07458.

The H-atoms were positioned geometrically (C–H = 0.93–0.96 Å) and refined as riding with *U*_iso_(H) = *xU*_eq_(C), where *x* = 1.5 for methyl and *x* = 1.2 for other H-atoms.

### Crystal data

**Table:**

C_11_H_9_NO_3_	*F*(000) = 424
*M**_r_* = 203.19	*D*_x_ = 1.412 Mg m^−^^3^
Monoclinic, *P*2_1_/*c*	Mo *K*α radiation, λ = 0.71073 Å
Hall symbol: -P 2ybc	Cell parameters from 1267 reflections
*a* = 12.7018 (15) Å	θ = 1.6–26.0°
*b* = 10.2689 (12) Å	µ = 0.10 mm^−^^1^
*c* = 7.4695 (8) Å	*T* = 296 K
β = 101.067 (7)°	Prism, light yellow
*V* = 956.16 (19) Å^3^	0.30 × 0.25 × 0.23 mm
*Z* = 4	

### Data collection

**Table:**

Bruker Kappa APEXII CCD diffractometer	1887 independent reflections
Radiation source: fine-focus sealed tube	1267 reflections with *I* > 2σ(*I*)
Graphite monochromator	*R*_int_ = 0.030
Detector resolution: 8.00 pixels mm^-1^	θ_max_ = 26.0°, θ_min_ = 1.9°
ω scans	*h* = −12→15
Absorption correction: multi-scan (*SADABS*; Bruker, 2005)	*k* = −12→9
*T*_min_ = 0.969, *T*_max_ = 0.977	*l* = −9→9
7388 measured reflections	

### Refinement

**Table:**

Refinement on *F*^2^	Primary atom site location: structure-invariant direct methods
Least-squares matrix: full	Secondary atom site location: difference Fourier map
*R*[*F*^2^ > 2σ(*F*^2^)] = 0.041	Hydrogen site location: inferred from neighbouring sites
*wR*(*F*^2^) = 0.112	H-atom parameters constrained
*S* = 1.01	*w* = 1/[σ^2^(*F*_o_^2^) + (0.051*P*)^2^ + 0.1053*P*] where *P* = (*F*_o_^2^ + 2*F*_c_^2^)/3
1887 reflections	(Δ/σ)_max_ < 0.001
137 parameters	Δρ_max_ = 0.11 e Å^−^^3^
0 restraints	Δρ_min_ = −0.19 e Å^−^^3^

### Special details

**Table:**

Geometry. All e.s.d.'s (except the e.s.d. in the dihedral angle between two l.s. planes) are estimated using the full covariance matrix. The cell e.s.d.'s are taken into account individually in the estimation of e.s.d.'s in distances, angles and torsion angles; correlations between e.s.d.'s in cell parameters are only used when they are defined by crystal symmetry. An approximate (isotropic) treatment of cell e.s.d.'s is used for estimating e.s.d.'s involving l.s. planes.
Refinement. Refinement of *F*^2^ against ALL reflections. The weighted *R*-factor *wR* and goodness of fit *S* are based on *F*^2^, conventional *R*-factors *R* are based on *F*, with *F* set to zero for negative *F*^2^. The threshold expression of *F*^2^ > σ(*F*^2^) is used only for calculating *R*-factors(gt) *etc*. and is not relevant to the choice of reflections for refinement. *R*-factors based on *F*^2^ are statistically about twice as large as those based on *F*, and *R*- factors based on ALL data will be even larger.

### Fractional atomic coordinates and isotropic or equivalent isotropic displacement parameters (Å^2^)

**Table:**

	*x*	*y*	*z*	*U*_iso_*/*U*_eq_	
O1	0.20496 (11)	0.44118 (13)	−0.18267 (18)	0.0604 (4)	
O2	0.08846 (11)	0.44793 (14)	0.1941 (2)	0.0692 (5)	
O3	0.28200 (11)	0.79126 (14)	0.0549 (2)	0.0724 (5)	
N1	0.20700 (11)	0.59539 (14)	0.11254 (19)	0.0438 (4)	
C1	0.29485 (14)	0.51130 (17)	0.1042 (3)	0.0447 (4)	
C2	0.29351 (15)	0.43286 (17)	−0.0476 (3)	0.0476 (5)	
C3	0.38002 (18)	0.35292 (19)	−0.0525 (3)	0.0619 (6)	
H3	0.3800	0.2986	−0.1522	0.074*	
C4	0.46654 (18)	0.3536 (2)	0.0903 (4)	0.0708 (7)	
H4	0.5250	0.3001	0.0854	0.085*	
C5	0.46810 (18)	0.4312 (2)	0.2383 (3)	0.0698 (7)	
H5	0.5271	0.4308	0.3338	0.084*	
C6	0.38151 (16)	0.5103 (2)	0.2455 (3)	0.0577 (5)	
H6	0.3818	0.5633	0.3465	0.069*	
C7	0.2079 (2)	0.3746 (2)	−0.3493 (3)	0.0789 (7)	
H7A	0.2684	0.4044	−0.3978	0.118*	
H7B	0.1430	0.3920	−0.4355	0.118*	
H7C	0.2142	0.2827	−0.3267	0.118*	
C8	0.11142 (15)	0.55756 (19)	0.1598 (2)	0.0478 (5)	
C9	0.04849 (15)	0.6775 (2)	0.1639 (3)	0.0556 (5)	
H9	−0.0208	0.6816	0.1871	0.067*	
C10	0.10532 (15)	0.7772 (2)	0.1297 (3)	0.0560 (5)	
H10	0.0840	0.8639	0.1280	0.067*	
C11	0.20922 (15)	0.72958 (18)	0.0941 (2)	0.0489 (5)	

### Atomic displacement parameters (Å^2^)

**Table:**

	*U*^11^	*U*^22^	*U*^33^	*U*^12^	*U*^13^	*U*^23^
O1	0.0631 (9)	0.0606 (9)	0.0594 (9)	0.0035 (7)	0.0164 (7)	−0.0146 (7)
O2	0.0603 (9)	0.0562 (9)	0.0953 (12)	−0.0116 (7)	0.0252 (8)	0.0111 (8)
O3	0.0643 (9)	0.0516 (9)	0.1068 (12)	−0.0081 (8)	0.0303 (9)	0.0103 (8)
N1	0.0435 (8)	0.0362 (8)	0.0552 (9)	−0.0021 (7)	0.0183 (7)	−0.0045 (7)
C1	0.0419 (10)	0.0386 (10)	0.0578 (12)	0.0002 (8)	0.0198 (9)	0.0032 (8)
C2	0.0498 (11)	0.0381 (10)	0.0600 (12)	0.0010 (9)	0.0232 (10)	0.0045 (8)
C3	0.0695 (14)	0.0451 (12)	0.0814 (15)	0.0089 (11)	0.0403 (13)	0.0056 (10)
C4	0.0571 (14)	0.0572 (15)	0.107 (2)	0.0192 (11)	0.0393 (14)	0.0293 (14)
C5	0.0532 (13)	0.0739 (16)	0.0824 (17)	0.0082 (12)	0.0137 (12)	0.0250 (14)
C6	0.0539 (12)	0.0576 (13)	0.0621 (13)	−0.0011 (10)	0.0125 (10)	0.0076 (10)
C7	0.0974 (18)	0.0750 (16)	0.0687 (15)	−0.0085 (14)	0.0271 (13)	−0.0231 (12)
C8	0.0437 (11)	0.0507 (12)	0.0504 (11)	−0.0071 (9)	0.0129 (9)	−0.0012 (9)
C9	0.0436 (10)	0.0657 (14)	0.0598 (12)	0.0055 (10)	0.0155 (9)	−0.0050 (10)
C10	0.0547 (12)	0.0471 (12)	0.0665 (13)	0.0090 (10)	0.0123 (10)	−0.0067 (9)
C11	0.0497 (11)	0.0431 (11)	0.0546 (11)	−0.0018 (9)	0.0121 (9)	−0.0012 (9)

### Geometric parameters (Å, º)

**Table:**

O1—C2	1.362 (2)	C4—H4	0.9300
O1—C7	1.426 (2)	C5—C6	1.377 (3)
O2—C8	1.203 (2)	C5—H5	0.9300
O3—C11	1.202 (2)	C6—H6	0.9300
N1—C8	1.383 (2)	C7—H7A	0.9600
N1—C11	1.386 (2)	C7—H7B	0.9600
N1—C1	1.422 (2)	C7—H7C	0.9600
C1—C6	1.371 (3)	C8—C9	1.471 (3)
C1—C2	1.388 (3)	C9—C10	1.307 (3)
C2—C3	1.378 (3)	C9—H9	0.9300
C3—C4	1.377 (3)	C10—C11	1.479 (3)
C3—H3	0.9300	C10—H10	0.9300
C4—C5	1.360 (3)		
			
C2—O1—C7	117.40 (17)	C1—C6—H6	119.9
C8—N1—C11	109.89 (15)	C5—C6—H6	119.9
C8—N1—C1	125.12 (15)	O1—C7—H7A	109.5
C11—N1—C1	124.64 (15)	O1—C7—H7B	109.5
C6—C1—C2	120.45 (18)	H7A—C7—H7B	109.5
C6—C1—N1	119.44 (17)	O1—C7—H7C	109.5
C2—C1—N1	120.10 (17)	H7A—C7—H7C	109.5
O1—C2—C3	124.53 (18)	H7B—C7—H7C	109.5
O1—C2—C1	116.59 (16)	O2—C8—N1	125.28 (18)
C3—C2—C1	118.9 (2)	O2—C8—C9	128.61 (18)
C4—C3—C2	119.9 (2)	N1—C8—C9	106.09 (16)
C4—C3—H3	120.1	C10—C9—C8	109.24 (17)
C2—C3—H3	120.1	C10—C9—H9	125.4
C5—C4—C3	121.2 (2)	C8—C9—H9	125.4
C5—C4—H4	119.4	C9—C10—C11	108.73 (17)
C3—C4—H4	119.4	C9—C10—H10	125.6
C4—C5—C6	119.4 (2)	C11—C10—H10	125.6
C4—C5—H5	120.3	O3—C11—N1	125.41 (17)
C6—C5—H5	120.3	O3—C11—C10	128.60 (18)
C1—C6—C5	120.3 (2)	N1—C11—C10	105.98 (16)
			
C8—N1—C1—C6	−100.8 (2)	N1—C1—C6—C5	−178.87 (17)
C11—N1—C1—C6	71.7 (2)	C4—C5—C6—C1	−0.3 (3)
C8—N1—C1—C2	80.5 (2)	C11—N1—C8—O2	−176.12 (18)
C11—N1—C1—C2	−107.0 (2)	C1—N1—C8—O2	−2.7 (3)
C7—O1—C2—C3	−8.0 (3)	C11—N1—C8—C9	2.25 (19)
C7—O1—C2—C1	172.00 (17)	C1—N1—C8—C9	175.70 (16)
C6—C1—C2—O1	−179.12 (16)	O2—C8—C9—C10	175.7 (2)
N1—C1—C2—O1	−0.4 (2)	N1—C8—C9—C10	−2.6 (2)
C6—C1—C2—C3	0.9 (3)	C8—C9—C10—C11	1.9 (2)
N1—C1—C2—C3	179.59 (15)	C8—N1—C11—O3	179.57 (19)
O1—C2—C3—C4	178.86 (17)	C1—N1—C11—O3	6.1 (3)
C1—C2—C3—C4	−1.1 (3)	C8—N1—C11—C10	−1.15 (19)
C2—C3—C4—C5	0.7 (3)	C1—N1—C11—C10	−174.64 (16)
C3—C4—C5—C6	0.1 (3)	C9—C10—C11—O3	178.7 (2)
C2—C1—C6—C5	−0.1 (3)	C9—C10—C11—N1	−0.5 (2)
